# Practicing functional nasal surgery in the non-urban setting: experience from a single center

**DOI:** 10.1093/jscr/rjac119

**Published:** 2022-04-23

**Authors:** Tristan Tham, Matthew I Saleem, McKenna Hawthorne, Alexandros Georgolios

**Affiliations:** Department of Otolaryngology, Donald and Barbara Zucker School of Medicine at Hofstra/Northwell, New York, NY, USA; Department of Otolaryngology, Donald and Barbara Zucker School of Medicine at Hofstra/Northwell, New York, NY, USA; Poplar Bluff Regional Medical Center, Poplar Bluff, MO, USA; Poplar Bluff Regional Medical Center, Poplar Bluff, MO, USA

## Abstract

Nasal airway obstruction is a prevalent chief complaint in the contemporary facial plastic surgery practice. Studies report an asymmetric distribution of plastic surgeons across the United States with a disproportionately high concentration of surgeons practicing in urban areas. The lack of elective specialist care creates unique challenges for these patients who may need to travel and dedicate time to reach a nasal surgery expert. We conducted a retrospective chart review to report our experience from practicing functional nasal surgery in such a non-urban setting in the United States. A total of 103 patients underwent functional nasal surgery (FNS) between May 2015 and August 2021 including septoplasty, inferior turbinate reduction, septorhinoplasty and nasal valve procedures. We present the epidemiological characteristics, surgical techniques used and postoperative complications and illuminate the unique characteristics of practicing FNS in the non-urban setting.

## INTRODUCTION

Nasal airway obstruction (NAO) is a prevalent chief complaint in the contemporary facial plastic surgery practice. Studies report an asymmetric distribution of plastic surgeons across the United States: a disproportionately high concentration of surgeons practice in urban areas [1]. Li *et al*. [2] identified urban location as an independent risk factor for subsequent septorhinoplasty following initial nasal bone fracture. This reflects the lack of elective specialist care access for these patients who may need to travel and dedicate time to reach a nasal surgery expert.

## CASE SERIES / METHODS

We conducted a retrospective chart review with the approval of the Ethics Committee of our institution. A total of 103 patients underwent functional nasal surgery (FNS) between May 2015 and August 2021 including septoplasty, inferior turbinate reduction, septorhinoplasty and nasal valve procedures (CPT codes 30 520, 30 802, 30 420, 30 465). All patients had failed previous management with at least 6 weeks of intranasal steroids. All surgical procedures were performed by the lead author.

## CASE SERIES/RESULTS

Sixty-four patients self-identified as males (62.1%) and 39 as females (37.9%). Ages ranged from 16 to 78 years (mean 38.05). Fifty-two patients (50.5%) reported a history of nasal trauma. Seven patients (6.8%) had previous FNS (reported septoplasty and/or inferior turbinate reduction) and 94 (91.2%) reported no previous nasal procedures. Forty-three patients (41.7%) were never smokers, 42 patients (40.8%) were active smokers and 18 patients (17.5%) reported past smoking history. Eighteen patients (17.5%) had a diagnosis of diabetes at the time of surgery (Table 1).

**Figure 1:**
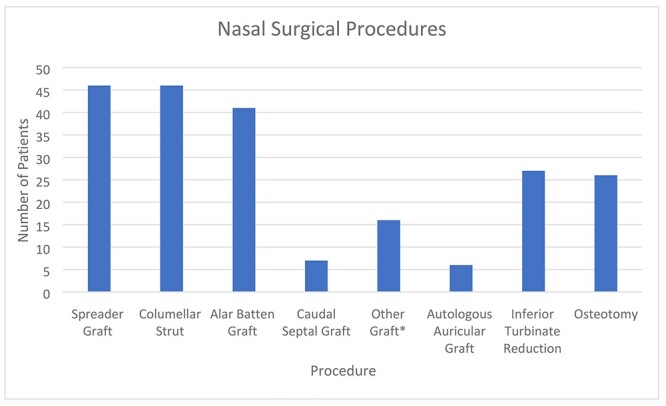
Nasal surgical procedures.

Spreader grafts were used at 46 patients (44.7%), columellar struts at 46 patients (44.7%), alar batten grafts at 41 patients (39.8%), caudal septal grafts at 7 patients (6.8%), other grafts reported at 16 patients (15.5%), autologous auricular graft harvested at 6 patients (5.8%) and inferior turbinate reduction performed at 27 (26.2%). Osteotomies were required in 26 patients (25.2%). Caudal septal grafts were reported when the L-strut required replacement (anterior septal reconstruction/extracorporeal septoplasty). Other grafts included on-lay grafts, alar rim grafts and lateral crura strut grafts (Fig. 1).

**Table 1:** Demographics of patients

**Characteristic**	**Percentage**
Gender	
Male	62.1
Female	37.9
Surgical history	
Previous nasal trauma	50.5
Previous nasal surgery	6.8
No previous nasal surgery	91.2
Smoking history	
Never smoker	41.7
Past smoking history	17.5
Active smoker	40.8
Type 2 diabetes mellitus	17.5

Six postoperative complications (5.8%) were reported in four patients (3.9%). These included three posterior septal perforations, two cases of severe postoperative bleeding, one infection and one case of minimal persistent bleeding that required medical attention. All septal perforations were asymptomatic. The infection manifested with minimal drainage in the mid-columellar incision and resolved with a course of antibiotics. The minimal postoperative bleeding occurred in a patient who was managed only with inferior turbinate reduction.

## DISCUSSION

Limited by the relatively small size, we report our experience from FNS in a non-urban center. About 50.4% of our patients reported history of nasal fracture, making post-traumatic FNS a major part of our practice; 40.7% of our patients were active smokers, and 17.4% reported past smoking history, numbers unparalleled to any rhinoplasty studies. A tertiary center study reported 11% active smokers and 30% previous smokers among patients undergoing nasoseptal surgery [3]. This difference may reflect higher incidence of tobacco use in our area compared with the national incidence of smoking in adults (14% in 2019) [4]. Our complication rate was 5.8%, similar to previously reviewed (bleeding-related complications 0.2–6.7%, infection 0–4%, septal perforations 0–2.9%) [5]. Two patients had a complicated postoperative course including both postoperative bleeding and subsequent septal perforations. Only 6.7% were revision cases, perhaps indicating the hardship in reaching expert care.

## CONCLUSION

Our results illuminate the unique characteristics of practicing FNS in the non-urban setting. Solid clinical expertise is required to manage these challenging patients.

## CONFLICT OF INTEREST STATEMENT

None declared.

## FUNDING

None.
